# Cy3-RgIA-5727 Labels and Inhibits α9-Containing nAChRs of Cochlear Hair Cells

**DOI:** 10.3389/fncel.2021.697560

**Published:** 2021-07-23

**Authors:** Fernando Fisher, Yuanyuan Zhang, Philippe F. Y. Vincent, Joanna Gajewiak, Thomas J. Gordon, Elisabeth Glowatzki, Paul Albert Fuchs, J. Michael McIntosh

**Affiliations:** ^1^Department of Biology, University of Utah, Salt Lake City, UT, United States; ^2^The Center for Hearing and Balance, Otolaryngology-Head and Neck Surgery, The Johns Hopkins University School of Medicine, Baltimore, MD, United States; ^3^George E. Wahlen Department of Veterans Affairs Medical Center, Salt Lake City, UT, United States; ^4^Department of Psychiatry, University of Utah School Medicine, Salt Lake City, UT, United States

**Keywords:** conotoxin, nAChR, cochlea, mouse, *Xenopus*

## Abstract

Efferent cholinergic neurons inhibit sensory hair cells of the vertebrate inner ear through the combined action of calcium-permeable α9α10-containing nicotinic acetylcholine receptors (nAChRs) and associated calcium-dependent potassium channels. The venom of cone snails is a rich repository of bioactive peptides, many with channel blocking activities. The conopeptide analog, RgIA-5474, is a specific and potent antagonist of α9α10-containing nAChRs. We added an alkyl functional group to the N-terminus of the RgIA-5474, to enable click chemistry addition of the fluorescent cyanine dye, Cy3. The resulting peptide, Cy3-RgIA-5727, potently blocked mouse α9α10 nAChRs expressed in *Xenopus* oocytes (IC_50_ 23 pM), with 290-fold less activity on α7 nAChRs and 40,000-fold less activity on all other tested nAChR subtypes. The tight binding of Cy3-RgIA-5727 provided robust visualization of hair cell nAChRs juxtaposed to cholinergic efferent terminals in excised, unfixed cochlear tissue from mice. Presumptive postsynaptic sites on outer hair cells (OHCs) were labeled, but absent from inner hair cells (IHCs) and from OHCs in cochlear tissue from α9-null mice and in cochlear tissue pre-incubated with non-Cy3-conjugated RgIA-5474. In cochlear tissue from younger (postnatal day 10) mice, Cy3-RgIA-5727 also labeled IHCs, corresponding to transient efferent innervation at that age. Cy3 puncta in Kölliker’s organ remained in the α9-null tissue. Pre-exposure with non-Cy3-conjugated RgIA-5474 or bovine serum albumin reduced this non-specific labeling to variable extents in different preparations. Cy3-RgIA-5727 and RgIA-5474 blocked the native hair cell nAChRs, within the constraints of application to the excised cochlear tissue. Cy3-RgIA-5727 or RgIA-5474 block of efferent synaptic currents in young IHCs was not relieved after 50 min washing, so effectively irreversible.

## Introduction

Mammalian nicotinic acetylcholine receptors (nAChRs) are pentameric ligand gated ion channels that are used for neurotransmission throughout the nervous system. The subunit combinations of these channels confer distinct pharmacological and biophysical properties that potentially allow for pharmacological discrimination (Jin et al., 2019). Cone snails are predatory marine organisms that employ complex mixtures of venom peptides. One function is to paralyze prey for capture, but it is increasingly apparent that venom components also are used to cause perceptual disorientation in prey or predators (Dutertre et al., 2014). Sensory hair cells are cilia-like structures that convert mechanical energy into electrical signals. Such hair cells are present in marine organisms and used to detect motion, pressure, and vibration of surrounding water (Fritzsch and Straka, 2014). Fish, for example, have hair cells arranged in a mechanosensory lateral line organ that is used, in part, to avoid capture. Recently it was demonstrated that these lateral line hair cells utilize α9 nAChRs at the efferent synapse to mediate ACh neurotransmission (Carpaneto Freixas et al., 2021).

One of the most conserved features of cone snail venoms are conotoxins that target nAChRs. Each venom examined has its own distinct complement of nAChR antagonists. A subset of these peptides have been shown to target α9-containing nAChRs selectively, including those present in mammalian cochlear hair cells (McIntosh et al., 2005; Luo et al., 2015). α-Conotoxin-RgIA, isolated from *Conus regius* is a specific and potent antagonist of α9-containing nAChRs as heterologously expressed in *Xenopus* oocytes as well as natively expressed α9α10 nAChRs (Ellison et al., 2006).

Efferent cholinergic neurons inhibit sensory hair cells through the combined action of calcium-permeable α9α10-containing nAChRs and associated calcium-dependent potassium channels (Glowatzki and Fuchs, 2000; Elgoyhen et al., 2009). Efferent synaptic contacts are made onto outer hair cells (OHCs) in the mature cochlea and onto inner hair cells (IHCs) prior to the onset of hearing, approximately the first two postnatal weeks in mice and rats. Considerable progress has been made defining the development, postsynaptic function, plasticity, and modulation of inner ear efferents. *In situ* hybridization has shown the expression of α9 mRNA in hair cells of the vertebrate inner ear (Elgoyhen et al., 1994; Hiel et al., 1996; Morley et al., 1998). Binding of fluorophore conjugated α-bungarotoxin (binds to a number of nAChR subtypes) has been used to show membrane localization of the functional receptor (Simmons and Morley, 1998; Roux et al., 2011). Recent success has been achieved by genetically tagging mouse α9 with visualized markers (Vyas et al., 2020), but this approach is not applied easily to other species. Therefore, in the absence of reliable antibodies, further progress would be improved by selective, potent biomarkers for α9-containing nAChRs. Thus, this work examines functional activity and labeling by a fluorophore conjugated analog of α-conotoxin RgIA (Cy3-RgIA-5727) of heterologously expressed and native α9α10-containing nAChRs. This has a twofold aim, to quantify binding and blocking by Cy3-RgIA-5727, and to determine to what extent the Cy3-conjugated RgIA analog retains its capacity to label and block α9-containing nAChRs in their native tissue, thereby confirming the utility of Cy3-conjugated analog of RgIA as a biomarker for these receptors.

## Materials and Methods

### Solid Phase Peptide Synthesis

RgIA-5727 was synthesized on 0.05 mmol scale using an Apex 396 automated peptide synthesizer (AAPPTec, Louisville, KY, United States), applying standard solid-phase Fmoc (9-fluorenylmethyloxycarbonyl) protocols Fmoc-Arg(Pbf)-Wang resin (0.3 mmol/g load; Peptides International). All standard amino acids, Fmoc-Pra-OH, Fmoc-Pen(Trt)-OH (Pen), Fmoc-beta-HTyr(tBu)-OH were purchased from AAPPTec except for Fmoc-3-iodo-L-Tyr-OH (I-3-Y) (Peptides International). Side-chain protection for the following amino acids was as follows: Arg, 2,2,4,6,7-pentamethyldihydrobenzofuran-5-sulfonyl (Pbf); Thr, Tyr, beta-HTyr, tert-butyl (tBu); Lys, tert-butyloxycarbonyl (Boc); Gln, trityl (Trt). To promote the correct folding of the disulfide bridge a trityl protected ^3^Pen residue was paired with ^12^Cys (Trt), while the other pair of Cys residues, ^2^Cys and ^8^Cys were Acm protected. Coupling activation was achieved with 1 eq of 0.4 M benzotriazol-1-yloxytripyrrolidinophosphonium hexafluorophosphate and 2 eq of 2 M *N*,*N*-diisopropylethyl amine in *N*-methyl-2-pyrrolidone as the solvent. For each coupling reaction, a 10-fold excess of amino acid was used, and the reaction was carried out for 60 min. For non-standard amino acids 5 fold excess was used and the coupling reaction was conducted for 90 min. Fmoc deprotection was performed for 20 min with 20% (v/v) piperidine in dimethylformamide.

### Peptide Cleavage and Purification

The peptide was cleaved from 90 mg resin using Reagent K consisting of trifluoroacetic acid/phenol/ethanedithiol/thioanisole/H_2_O (9:0.75:0.25:0.5:0.5 by volume). Next, the cleavage mixture was filtered and precipitated with 150 mL of cold methyl-tert-butyl ether (MTBE). The crude peptide was then precipitated by centrifugation at 7,000 × *g* for 7 min and washed twice with 150 mL cold MTBE. The crude peptide was diluted with 50 mL of the HPLC buffer B and purified by reversed-phase (RP) HPLC using preparative C18 Vydac column (218TP101522, 250 mm × 22 mm, 5-μm particle size), eluted with a linear gradient ranging from 10 to 50% buffer B in 40 min at a flow rate 20 mL/min. The HPLC buffers were 0.1% (vol/vol) trifluoroacetic acid in water (buffer A) and 0.092% trifluoroacetic acid (vol/vol) in 60% aqueous acetonitrile (vol/vol) (buffer B). The eluent was monitored by measuring absorbance at 220/280 nm. Purity of the peptide was assessed by analytical C18 Vydac RP-HPLC (218TP54, 250 mm × 4.6 mm, 5-μm particle size) using the same gradient as described above with a flow rate 1 mL/min. Out of 90 mg resin cleaved 2,130 nmol of linear RgIA-5727 was prepared (43% purity).

#### First Disulfide Bond Formation (^4^Pen-^13^Cys)

Linear RgIA-5727 (2,130 nmol) was dissolved in 150 mL buffer A and was added drop-wise to a solution consisting of 20 mM potassium ferricyanide (0.659 g) and 0.1 M Tris base (1.21 g) in 100 mL of H_2_O (peptide final concentration was approximately 20 μM and the pH was 7.5). The reaction was carried out for 45 min at room temperature and then terminated by acidifying the solution with 250 mL buffer A. The reaction mixture was then passed through a disposable C18 cartridge (Thermo Fisher, HyperSep Spe C18 1,000 mg/8 mL), and the peptide was eluted using buffer B. The efficiency of the reaction as well as purity of the peptide was analyzed by analytical RP-HPLC as described for the linear peptide. The monocyclic RgIA-5727 was obtained in 60% yield and with 63% purity.

#### Second Disulfide Bond Formation (^3^Cys–^9^Cys)

Simultaneous removal of the acetamidomethyl groups and closure of the second disulfide bridge was accomplished by iodine oxidation. Seventy-six milligrams of iodine (2 mmols) was added to 15 mL of acetonitrile and stirred until completely dissolved. Then, 45 mL of H_2_O was added followed by 1.8 mL of trifluoroacetic acid. The monocyclic RgIA-5727 solution of 1,270 nmol diluted with 90 mL buffer A, was dripped into 60 mL of the 10 mM iodine solution and allowed to react for 10 min at room temperature. Peptide final concentration was kept close to 20 μM. The reaction was quenched by adding 5–10 drops of 1 M freshly prepared ascorbic acid (0.176 g, 1 mmol) solution in water (1 mL) until the reaction mixture became transparent. The reaction was then diluted fivefold with buffer A and subsequently purified by RP-HPLC using preparative C18-column as described for the linear peptide to obtain 1,030 nmol of the fully folded RgIA-5727. Purity (99%) and final yield (48%) of RgIA-5727 was determined by RP-HPLC using analytical C18 column using the gradient described earlier for the linear peptide. The calculated mass of [MH]^+1^ = 1983.27 Da was verified to be [M+H]^+1^ = 1982.72 Da by matrix-assisted laser desorption ionization time-of-flight mass spectrometry (MALDI-TOF) at the Mass Spectrometry and Proteomics Core Facility at the University of Utah.

#### Copper Assisted 1,3-Dipolar Cycloaddition Reaction

Cy3 azide (Cy3) and RgIA-5727 were conjugated in a copper assisted azide–alkyne 1,3-dipolar cycloaddition reaction, to generate Cy3-1,2,3-triazole-RgIA-5727 (Cy3-RgIA-5727). Before starting the reaction, freshly prepared reagent stocks (excluding Cy3 azide) were treated with argon gas for 10 min. Concentration of the stock solutions are given in parentheses. To 2 μL of tris[(1-hydroxy-propyl-1H-1,2,3-triazole-4-yl)methyl]amine (THPTA, 36 mM) in anhydrous DMSO, 2 μL of CuS0_4_ solution (36 mM) was added and the reaction was mixed for 5 min. Next 2 μL of 72 mM ascorbic acid was added to the reaction mixture to reduce Cu (II) to Cu (I). The mixture was reacted for 5 min at room temperature, followed by addition of 3 μL of Cy3 azide (50 mM) in anhydrous DMSO and 6 μL of RgIA-5727 (8.3 mM) in 50% aqueous acetonitrile. The reaction tube was then vortexed gently, flushed with argon and reacted for 24 h at 50°C. The reaction solution was quenched with 5 μL ethylenediamine tetraacetic acid (EDTA, 0.2 M) in water and diluted with buffer A to 20% acetonitrile final concentration for purification. Cy3-RgIA-5727 was purified with an analytical C18 Vydac RP-HPLC (218TP54, 250 mm × 4.6 mm, 5-μm particle size) using a linear gradient ranging from 2 to 100% buffer B over 50 min at a flow rate of 2 mL/min. The eluent was monitored by measuring absorbance at 220/280/555 nm. Cy3-RgIA-5727 was calculated by absorption spectroscopy at excitation max 555 nm using the extinction coefficient (ε) 150,000 M^–1^ cm^–1^. Purchased reagents include CuS0_4_ (Sigma Aldrich, St. Louis, MO, United States), THPTA (Click Chemistry Tools, Scottsdale, AZ, United States), ascorbic acid (Sigma Aldrich), aminoguanidine (Sigma Aldrich), Cy3 azide (catalog 3 A1030, Lumiprobe, Hunt Valley, MD, United States), and EDTA (Sigma Aldrich).

### Two-Electrode Voltage-Clamp Recording

All procedures involving frogs were approved by the University of Utah Animal Care and Use Committee (Protocol #20-07003). *Xenopus laevis* (Xenopus1, Dexter, MI, United States) oocytes were used to heterologously express cloned mouse nAChR subtypes (Christensen et al., 2017). Recordings were made 1–5 days post-injection. Oocytes were voltage-clamped at − mV at room temperature and pulsed for 1 s, every 60 s, with a bolus of acetylcholine; RgIA analogs were applied as described previously (McIntosh et al., 2005; Hone et al., 2021).

### Mice

All procedures involving animals were approved by the Johns Hopkins University Animal Care and Use Committee (Protocol #MO19M478). Mice were housed in the Johns Hopkins Research Animal Resource Center where they had 24-h access to food and water. Mouse pups of either sex were deeply anesthetized (isoflurane or CO_2_ inhalation) until unresponsive to toe or tail pinch, then decapitated. C57BL/6J mice were obtained from The Jackson Laboratory (#000664). Mice lacking functional α9 nAChRs (α9-null, C57Bl6 background) were custom generated in a breeding colony established at Johns Hopkins, and have been characterized previously (Vetter et al., 1999). ChAT-Cre mice (B6.129S-*Chattm1(cre)Lowl*/MwarJ, stock #031661) and ReaChR-mCitrine (B6.Cg-*Gt(ROSA)26Sor^*tm2.2Ksvo*^*/J, stock #026294) were obtained from Jackson Labs. Both males and females were randomly chosen from designated litters for tissue removal to be used in histological or electrophysiological assays. No other randomization was performed to allocate subjects in the study.

### Cochlear Histology

Segments of the apical and middle turns (1.0–1.5 mm in length) were excised from unfixed cochleas of postnatal day nine to fourteen day old (P9 to P14) C57/Bl6 or α9-null mice (C57 background) for confocal microscopy. The otic capsule was dissected from the temporal bone and the bony shell delicately chipped away with blunted forceps (Dumont #5). The lateral wall and Reissner’s membrane were cut away, and the tectorial membrane gently removed with fine forceps (Dumont #55). An apical or middle cochlear turn was obtained by cutting through the cochlear modiolus with iridectomy scissors. An isolated turn was then transferred to a coverslip to which fine pins (*minuten nadeln*) were glued, leaving most of the tip free. This acted as a spring clip under which the cochlear segment was secured. The cover slip with tissue attached was transferred to a four-well petri dish, where each well was pre-filled with 100 μL of 10 nM Cy3-RgIA-5727 or non-conjugated RgIA-5474 (Gajewiak et al., 2021) in extracellular saline. In later experiments, 0.1 mg/ml bovine serum albumin (BSA) was included to reduce non-specific absorption of conopeptide. Tissue was incubated with 10 nM Cy3-RgIA-5727 or non-conjugated RgIA-5474, for 15 min, then transferred to a shallow chamber filled with extracellular saline on the stage of the confocal microscope. The tissue was washed by perfusion of extracellular saline at a rate of 1–3 ml/min to remove unbound peptide, and to maintain tissue health. Higher concentrations of Cy3-RgIA-5727 (up to 270 nM) were used for later experiments on ChAT Cre mice. The tissue also was exposed to FM4-64 (10–50 μM depending on the degree of tissue loading in each instance) that accumulates in and provides visualization of hair cells, as used previously (Roux et al., 2011). The labeled tissue was examined on a Nikon A1R-MP upright confocal microscope and *z*-stack image series acquired using Nikon Nis Element software. All 3-D videos were made with the *Movie Maker* tool in Nis Element software. Image analysis was conducted by a blinded observer in 3-D using a virtual reality viewing system (Syglass, Istovisio, Inc.). A second blinded observer recounted a subset of images for comparison. Cy3 puncta contacting FM4-64 positive hair cells were counted in nine cochlear segments containing on average 13 (±4, SD) IHCs and 44 (±15, SD) OHCs.

### Cellular Electrophysiology

Intracellular, gigaohm-seal recordings were made from IHCs in the apical cochlear turn excised from pre-hearing (P9–P11) mice. Hair cells were visualized using a 40× water immersion objective and differential interference contrast optics. Piezo-electric manipulators (Sutter Instruments ROE 200 and MP 285) positioned the recording pipette on a targeted hair cell. Recording pipettes were pulled from 1 mm borosilicate glass capillaries (World Precision Instrument “Kwik-Fil^TM^” #1B100F-4), fire-polished to resistances of 3–6 MΩ when filled with internal solution (mM): 135 KCl, 3.5 MgCl_2_, 0.1 CaCl_2_, 5 EGTA, 5 HEPES, and 2.5 Na_2_-ATP, pH 7.2, 290–310 mOsm, adjusted with KOH. Electrode series resistance ranged from 7 to 20 MΩ and was not compensated for these small synaptic currents. Tissue was continuously perfused with external saline (mM): 5.8 KCl, 144 NaCl, 1.3 CaCl_2_, 0.9 MgCl_2_, 0.7 NaH_2_PO_4_, 5.6 D-glucose, and 10 HEPES (300 mOsm, pH 7.4, adjusted with NaOH). Experimental solutions were perfused into the recording chamber by switching reservoirs of the gravity-fed perfusion system. Cholinergic synaptic currents were triggered by electrical shocks to the efferent neurons innervating young IHCs (Goutman et al., 2005). Shocks were delivered between a wire lead inserted into a 4–10 μm diameter glass electrode filled with external saline, and a return lead in the bath. The stimulating electrode was positioned 20 μm from the recorded cell. Shocks (typically 150 μV) 200 μs long were presented as 1 Hz trains. Cy3-RgIA-5727 or non-conjugated RgIA-5474 was bath perfused while prolonged trains of efferent shocks were used to evoke postsynaptic currents that were averaged to evaluate blocking efficacy. Recordings were conducted at room temperature.

A Multiclamp 700B amplifier (Axon Instruments) recorded membrane currents in voltage-clamp that were analyzed using pClamp (Axon, RRID:SCR_011323), MiniAnalysis (Synaptosoft, RRID:SCR_002184), GraphPad Prism 4 (GraphPad Software, RRID:SCR_002798), and Excel (Microsoft) software. Voltage command potentials are reported as membrane potential, ignoring the junction potential (−4 mV).

### Statistics, Rigor, and Reproducibility

Control and experimental results (presented in text as mean plus and minus standard deviation) were compared for significance using unpaired *t*-tests assuming unequal variance or ANOVA for multiple comparisons. Testing for normality was not conducted for these large effect sizes (i.e., efficacy of block) and small sample sizes. In addition to analysis by the original experimenter, “blinded” analysis of results was carried out by one or more observers not involved in conduct of the experiments. Initial attempts found that Cy3-RgIA-5727 labeled hair cells in the mouse cochlea, the main object of these studies of native receptors. Thus, no attempt was made to predetermine sample size so no sample calculation was performed. Labeling with Cy3-RgIA-5727 was performed on living (unfixed) tissue. Microdissection of fragile cochlear tissue from the hardened temporal bone is not always perfect, thus preparations with substantial damage and missing sensory epithelium were excluded from study. Functional block of inhibitory cholinergic synaptic transmission by peptide was slower than anticipated but reproducible in every recording lasting longer than 10 min. Synaptic blockade by Cy3-RgIA-5727 or RgIA-5474 required more than 10 min to take effect and was irreversible, thus, no primary or secondary endpoints were pre-specified. Quality control of intracellular recordings from hair cells in these experiments involved monitoring “leak current” at the standard holding voltage of −60 mV. A rapid increase in the resting conductance (“leak current”) signaled cell disruption.

## Results

### Synthesis of Cy3-RgIA-5727

**FIGURE 1:**
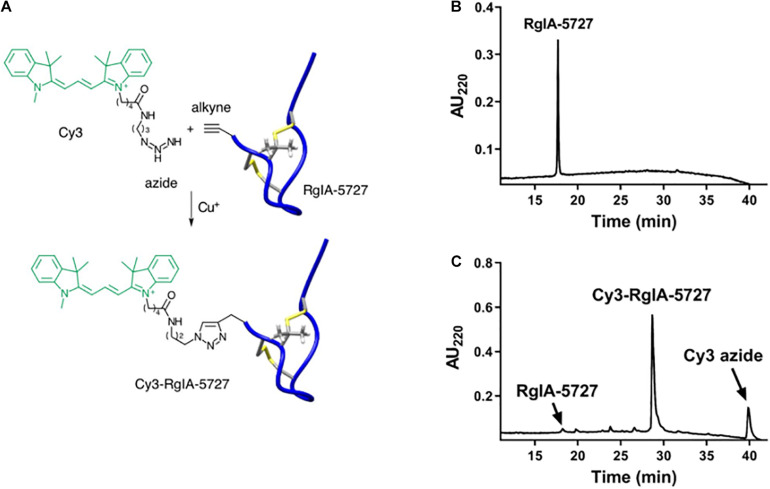
Synthesis and HPLC purification of Cy3-RgIA-5727. **(A)** The N-alkyne-containing RgIA analog was reacted with Cy3-azide, in the presence of Cu (I) as a catalyst, to form Cy3-RgIA-5727. **(B)** Chromatography profile of alkyne-bearing precursor RgIA-5727. **(C)** Chromatography profile of the crude click reaction mixture. The major peak corresponds to the desired product, Cy3-RgIA-5727.

Click chemistry involves a modular approach for the derivatization of template ligands. Molecular units can be joined in efficient, building-block fashion (Kolb et al., 2001; Ahmad Fuaad et al., 2013). We utilized this methodology to add a fluorescent reporter group to a high affinity peptide ligand of α9α10 nAChRs. The precursor RgIA-5727, L-propargylglycine (Pra)-containing analog of RgIA-5474 (Gajewiak et al., 2021), was prepared by solid-phase synthesis as described in Section “Materials and Methods,” with a Pra residue incorporated at the N-terminus of the peptide. The calculated mass [M + H]^+1^ is 1983.27 and was measured to be [M + H]^+1^ = 1982.72 by matrix-assisted laser desorption ionization time-of-flight mass spectrometry (MALDI-TOF). RgIA-5727 was conjugated with the fluorophore Cy3 in a copper (I) azide–alkyne 1,3-dipolar cycloaddition reaction (Figure 1A). The Cy3-RgIA-5727 was obtained in 78% yield (Figure 1B) and purified to >95% purity by RP-HLC. The calculated mass of Cy3-RgIA-5727 [MH]^+1^ = 2522.46 Da was verified to be 2522.13 Da by MALDI-TOF (Figure 1C).

**FIGURE 2:**
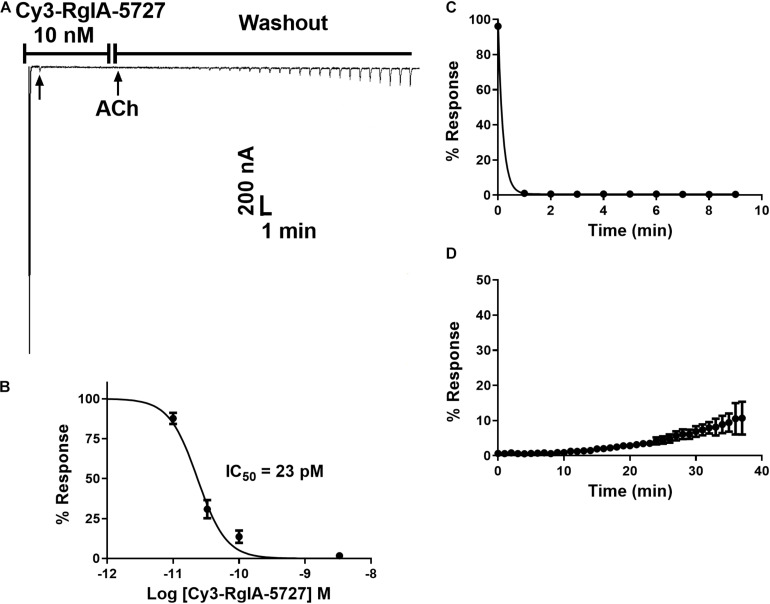
Block and recovery from block of α9α10 nAChRs by Cy3-RgIA-5727. **(A)** Cy3-RgIA-5727 (10 nM) was applied to *Xenopus* oocytes expressing mouse α9α10 nAChRs as described in Section “Materials and Methods.” Recovery from block after removal of peptide is shown. **(B)** Concentration response analysis indicated an IC_50_ of 23 pM (see Table 1); *n* = 3–4 oocytes. **(C)** Time course of block and **(D)** un-block by 10 nM Cy3-RgIA-5727; *n* = 3 oocytes.

**FIGURE 3:**
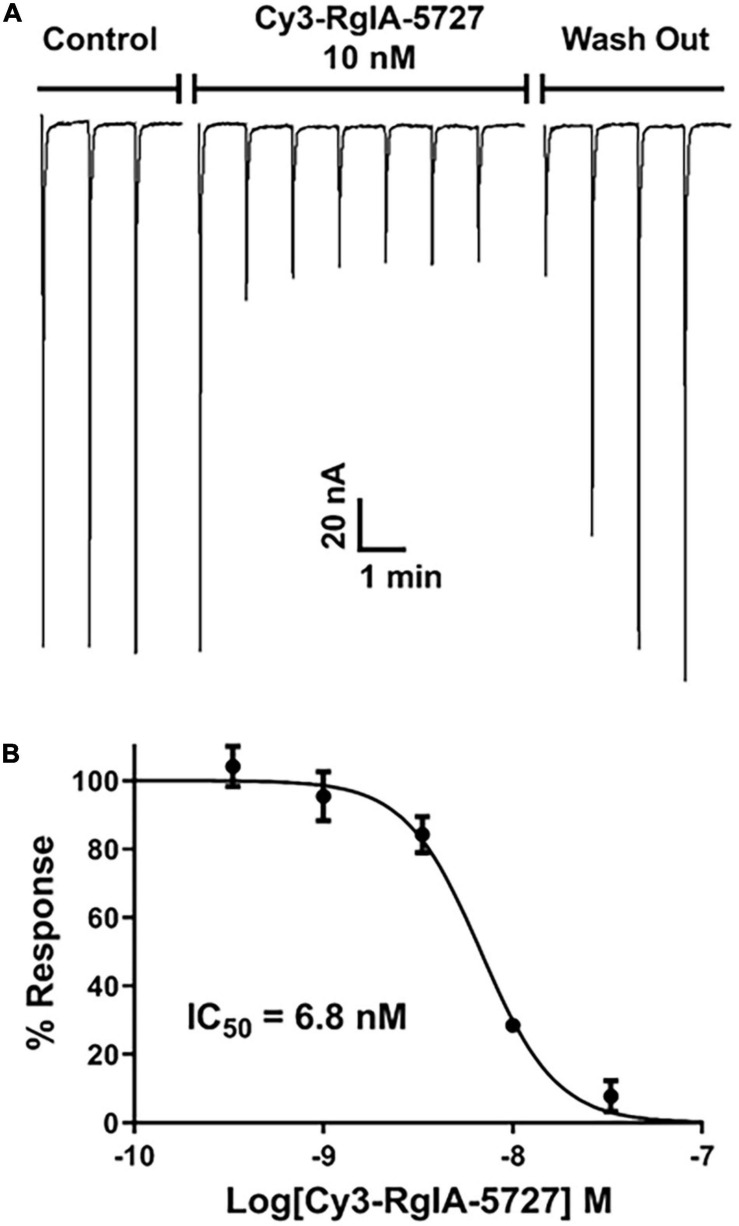
Block and recovery from block of α7 nAChRs by Cy3 RgIA-5727. **(A)** Cy3-RgIA-5727 (10 nM) was perfusion applied to *Xenopus laevis* oocytes expressing mouse α7 nAChRs. Note the rapid recovery from block after removal of peptide. **(B)** Concentration response analysis indicated an IC_50_ of 6.8 nM (see Table 1).

Functional activity was assessed by testing the Cy3-RgIA-5727 derivative on mouse α9α10 nAChRs heterologously expressed in *Xenopus* oocytes. Cy3-RgIA-5727 potently blocked the receptor in concentration-dependent manner with an IC_50_ of 23 pM (Figures 2A–C). Recovery from block following removal of peptide was slow, with <1% recovery at 5 min (Figures 2A,D). Subtype selectivity was assessed by measuring the activity at other nAChR subtypes. The next most potently blocked receptor was the α7 nAChR (Figure 3A), with an IC_50_ of 6.8 nM (Figure 3B and Table 1), 296-fold less potent than the α9α10 nAChR. Block of the two receptors could be distinguished readily by off-rate kinetics. In contrast to the very slowly reversible block of α9α10 nAChRs, recovery from the block of α7 nAChRs was rapid >99% recovery after 5 min (Figure 3A). This difference in off-rate kinetics allows for efficient washout from any non-α9α10 nAChRs. Block at all other subtypes was <50% at 1 μM concentration indicating >40,000-fold selectivity for the α9α10 nAChR (Table 1).

**TABLE 1:** Cy3-RgIA-5727 IC_50_ on *Xenopus* oocytes expressing nAChRs.

Subtype	IC50 (nM)	95% CI (nM)
α9α10	0.023	0.020–0.028
α7	6.8	5.8–7.9
α2β2	>1,000	
α2β4	>1,000	
α3β2	>1,000	
α3β4	>1,000	
α4β2	>1,000	
α4β4	>1,000	
α6/α3β2β3	>1,000	
α6/α3β4	>1,000	
α1β1δε	>1,000	

### Cy3-RgIA-5727 Labeling of the Mouse Cochlea

**FIGURE 4:**
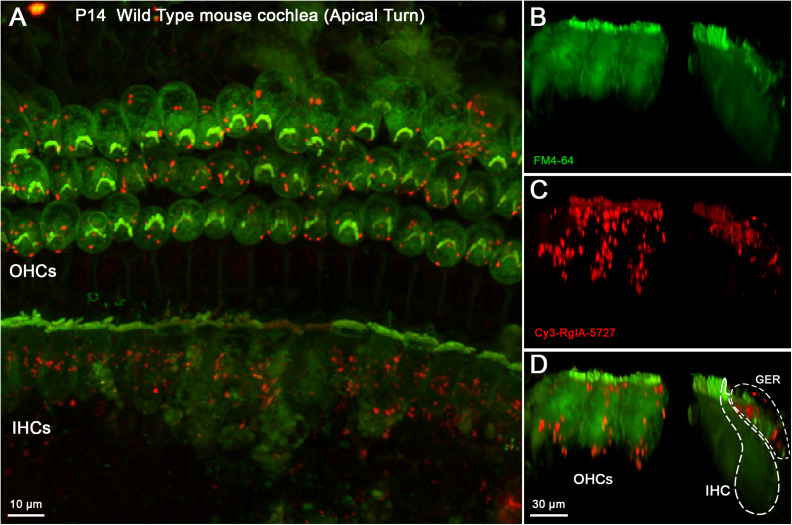
Cy3-RgIA-5727 label in Wild Type P14 mouse cochlea. At this age efferent synapses are found on OHCs with little to none of the transient efferent innervation to IHCs. Cy3-RgIA-5727 (10 nM) produced red puncta associated with OHCs (labeled green by accumulated FM dye). **(A)** Maximum intensity projection (MIP) from the apical surface of the cochlea. **(B)** Optical yz-plane view of cochlea (MIP), three rows of OHCs on left, IHCs on right, green FM4-64 accumulation fills hair cell somata and stereocilia. Note that the MIP shows multiple cells in each position. **(C)** Cy3-RgIA-5727 in yz-plane view. Large number of puncta on OHCs captured by MIP that includes approximately 10 hair cells in each position. FM4-64 fluorescence bleeds through to label stereocilia faintly. Additional puncta observed modiolar to the IHCs in region of greater epithelial ridge, but not along the synaptic pole of the IHCs. **(D)** Merged view of FM4-64 and Cy3-RgIA-5727 (MIP). Note that red Cy3 puncta do not have corresponding green puncta in panel **(B)**. Inner hair cell (IHC) and greater epithelial ridge (GER) outlined with dotted lines. Images exemplify results obtained from four cochleas from four mice. See Supplementary Video 1.

The Cy3-conjugated analog of RgIA-5727 produced localized puncta within the apical and middle turns of the cochlear epithelium (basal turns were not examined). In the P14 mouse cochlea, Cy3 puncta were observed on OHCs, where the majority of efferent synaptic contacts are found (Figure 4A). Puncta also were seen near the cuticular plate of OHCs (Figures 4C,D), where efferent synaptic contacts have been described in electron micrographic studies (Liberman et al., 1990; Nadol and Burgess, 1994). Note that these maximum intensity projections “sum” puncta from approximately 10 hair cells at each position, giving an impression of large numbers in a single cell. As seen in the whole mount views (Figure 4A), only 2–5 Cy3 puncta are associated with each OHC. Little or no punctate labeling was found at the synaptic pole of IHCs in P14 cochleas (Figures 4C,D), although Cy3 puncta were seen in the adjacent greater epithelial ridge (Supplementary Video 1).

**FIGURE 5:**
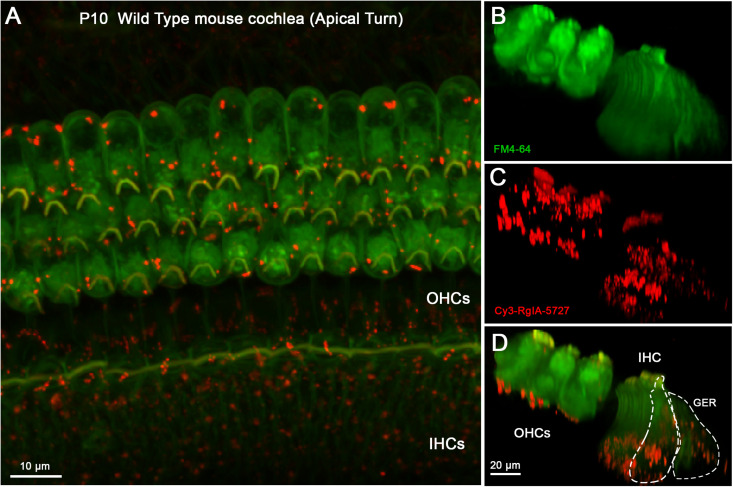
Cy3-RgIA-5727 label in Wild Type P10 cochlea. This age is near the peak of MOC innervation onto IHCs, and innervation of OHCs is well under way. **(A)** Maximum intensity projection (MIP) from the apical surface of the cochlea. **(B)** Optical yz-plane view of cochlea (MIP), three rows of OHCs on left, single row of IHCs on right, green FM4-64 accumulation fills multiple hair cells seen in depth. **(C)** Cy3-RgIA-5727 in yz-plane view (MIP); red puncta observed on OHCs and IHCs. **(D)** Merged view of FM4-64 and Cy3-RgIA-5727 (MIP). Red Cy3 puncta readily distinguished against green FM4-64 background. Inner hair cell (IHC) and greater epithelial ridge (GER) outlined with dashed lines. Images exemplify results from 12 cochleas from 12 mice. See Supplementary Video 2.

In younger cochlear segments (P10) Cy3 puncta were found at the synaptic pole of both IHCs and OHCs (Figure 5 and Supplementary Video 2). Multiple clusters of Cy3 puncta were observed at the synaptic pole of IHCs (Figures 5C,D), a pattern of labeling that was clearly missing at P14, after efferent synapses are lost. Cy3 puncta were visible in the greater epithelial ridge at both P10 (Figures 5C,D) and P14.

Cy3-RgIA-5727 was applied to unfixed tissue, necessitating a vital dye, FM4-64, for visualizing hair cells. Since the emission spectra of FM4-64 and Cy3 overlap, Cy3 images can include “background” FM4-64 fluorescence in the stereociliary bundle and cytoplasm. Nonetheless, discrete Cy3 puncta were readily distinguished from that background (e.g., Figures 4D, 5D), and there were no equivalent bright green puncta in the images where only FM4-64 was imaged (Figures 4B, 5B).

**FIGURE 6:**
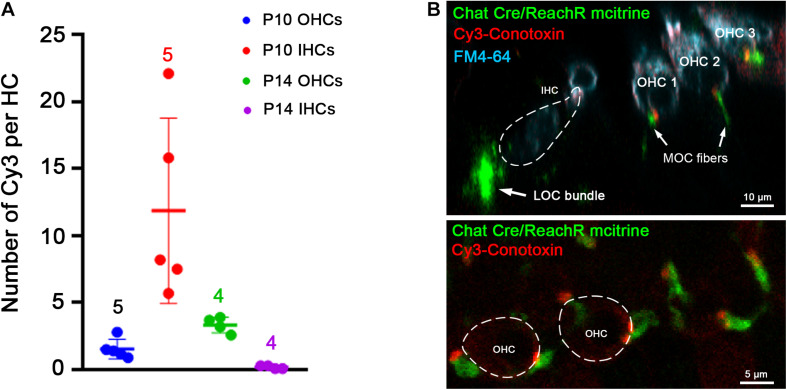
Cochlear location of Cy3-RgIA-5727 puncta. **(A)** Cy3 puncta associated with hair cells were counted in cochleas from C57 Bl6 mice before (P10) and after the onset of hearing (P14). **(B)** Cochleas from P23 mice expressing the fluorescent protein m-citrine under the ChAT promoter (Chat-Cre) were excised and exposed to Cy3-RgIA-5727 in the presence of 0.1 mg/ml BSA. Cy3-puncta (red) were found under OHCs, but not under IHCs. Note also the absence of Cy3 puncta medial to the IHCs. Cy3-puncta were closely aligned with m-citrine positive efferent terminals (green). See Supplementary Video 3 for 3-D view.

Punctate Cy3 labeling appeared in positions consistent with presumptive synaptic sites. Cy3 puncta were counted in middle turns of cochleas from young mice before and after the age of hearing onset (postnatal day 12–P12). This was done to determine whether labeling corresponds to known changes of efferent innervation around that time. Prior to the onset of hearing, efferent synapses are formed temporarily on IHCs as OHC innervation develops. After the onset of hearing, IHC synapses diminish as OHC innervation increases. From five P10 cochleas (pre-hearing-onset) including 174 OHCs and 53 IHCs, the average number of Cy3 puncta per OHC was 1.6 ± 0.7 (Figure 6A). A significantly greater number of puncta (11.9 ± 6.9, two-tailed unpaired *t*-test, *p* = 0.0105, *t* = 3.321, df = 8) were associated with IHCs at P10 compared to OHCs, as predicted by the robust efferent innervation of IHCs prior to the onset of hearing (Roux et al., 2011). In four cochleas from P14 mice including 179 OHCs and 54 IHCs, the number of OHC-associated Cy3 puncta was 3.4 (±0.6), significantly more than at P10 (two-tailed unpaired *t*-test, *p* = 0.005, *t* = 4.072, df = 7). Efferent synapses are lost from IHCs after the onset of hearing and there were significantly fewer Cy3 puncta per IHC at P14 (1.9 ± 0.9), compared to P10 (11.9 ± 6.9, *p* = 0.025, *t* = 2.84, df = 7, two-tailed unpaired *t*-test).

Thus, Cy3-fluorescent puncta of outer and IHCs were in positions predicted by developmental changes in patterns of efferent innervation. To confirm that association, Cy3-RgIA-5727 was applied to cochlear tissue excised from two P23 ChAT-Cre-ReachR mice expressing fluorescent m-citrine that is visible in cholinergic olivocochlear efferents in unfixed tissue. In these tissues, there was correspondence between Cy3 puncta and the m-citrine labeled terminals of efferent neurons on OHCs (Figure 6B and Supplementary Video 3). Note also that cholinergic efferent neurons beneath IHCs had no associated Cy3 puncta, consistent with absent α9 expression (Simmons, 2002; Katz et al., 2004). These are lateral olivocochlear (LOC) efferents that make cholinergic synapses onto Type I afferent terminals mediated by non-α9-containing nAChRs.

**FIGURE 7:**
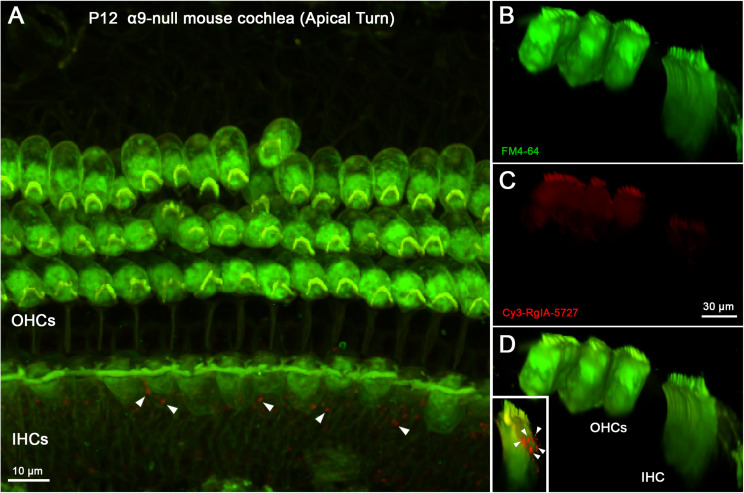
Cy3-RgIA-5727 labeling in P12 α9-null mouse cochlea. At this age efferent synapses occur on IHCs and OHCs, even in the absence of the α9 subunit. **(A)** Maximum intensity projection (MIP) from the apical surface of the cochlea. **(B)** Optical yz-plane view of cochlea (MIP), three rows of OHCs on left, IHCs on right, green FM4-64 accumulation. **(C)** Cy3-RgIA-5727 in yz-plane view (MIP). **(D)** Merged view of FM4-64 and Cy3-RgIA-5727 (MIP). No Cy3 puncta are found in the synaptic regions of IHCs or OHCs. Cy3-RgIA-5727 puncta occurred medial to the IHCs in the greater epithelial ridge [inset and white arrowheads in panel **(A)**]. Images exemplify results obtained from two cochleas from two knockout mice. See Supplementary Video 4.

Two control experiments were conducted to distinguish specific from non-specific labeling by Cy3-RgIA-5727. The α9 subunit is completely absent from cochlear hair cells in homozygous genetic knockout (α9 null) mice (Vetter et al., 1999) and no punctate synaptic Cy3-RgIA-5727 labeling was found on outer or IHCs in cochlear segments from P12 α9 null mice (Figure 7). Cy3 puncta within the greater epithelial ridge remained (Figures 7A,D inset), demonstrating that these do not result from expression of α9. BSA was not included in the perfusate for these experiments (Supplementary Video 4).

**FIGURE 8:**
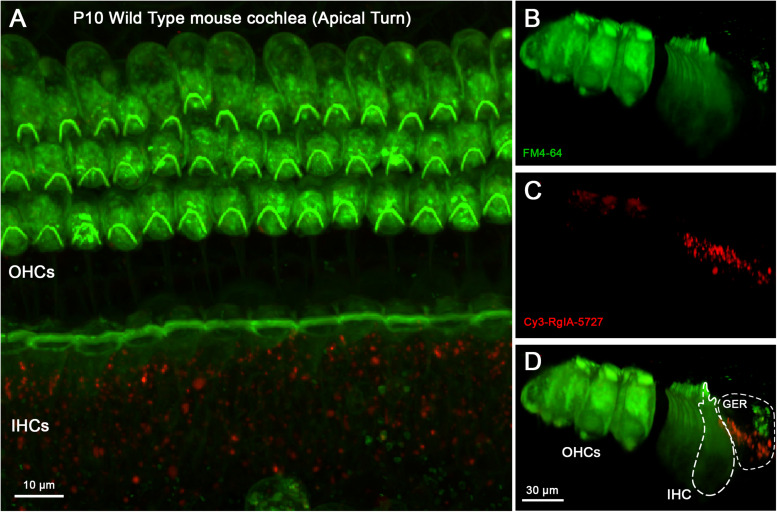
Cy3-RgIA-5727 labeling in P10 WT mouse cochlea pre-exposed to non-conjugated RgIA-5474. At this age one can expect efferent synapses on IHCs and OHCs. **(A)** Maximum intensity projection (MIP) from the apical surface of the cochlea. **(B)** Optical yz-plane view of cochlea (MIP), three rows of OHCs on left, single row of IHCs on right, green FM4-64 accumulation. **(C)** Cy3-RgIA-5727 in yz-plane (MIP). **(D)** Merged view of FM4-64 and Cy3-RgIA-5727 (MIP). No Cy3 puncta are found in the synaptic regions of IHCs (outlined with dashed lines) or OHCs but Cy3 puncta are seen in the greater epithelial ridge (GER, outlined with dashed lines). Images exemplify results obtained from two cochleas from two mice. See Supplementary Video 5.

To distinguish patterns of labeling further, wild type cochleas were pre-absorbed with unconjugated RgIA-5474 prior to exposure to Cy3-RgIA-5727. Under these conditions, no punctate Cy3 labeling was found on outer or IHCs in P10 cochleas (Figure 8). Cy3 puncta remained present in the greater epithelial ridge (Figure 8D) for these trials without BSA in the perfusate (Supplementary Video 5).

### Either Cy3-RgIA-5727 or RgIA-5474 Block Efferent Synaptic Currents in Young Inner Hair Cells

**FIGURE 9:**
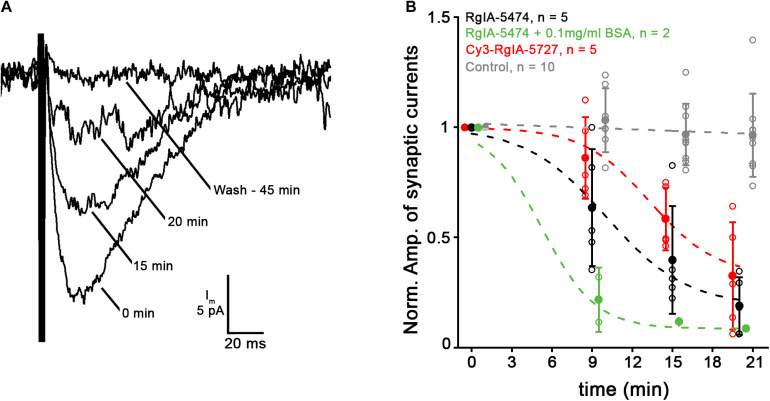
Application of 10 nM Cy3-RgIA-5727 or RgIA-5474 gradually reduced the amplitude of efferent postsynaptic currents. **(A)** Synaptic current recorded at –90 mV in a P9 IHC during a 1 Hz train of 100 shocks. Each response is the average response at the indicated time point as Cy3-RgIA-5727 spread through the tissue. After washing out toxin for 45 min there was no recovery. Recordings exemplify results obtained from five IHCs from five mice. **(B)** Reduction of efferent postsynaptic current (normalized to initial pre-block value) over time in the presence of 10 nM RgIA-5474 (black) or 10 nM Cy3-RgIA-5727 (red). All data points collected at 9, 15, 20 min but displaced horizontally for clarity. Experimental data fitted with $y=\frac{A_{1}-A_{2}}{1+e^{\left(x-x_{0}\right)/\text{dx}}}+A_{2}$, where *A*_1_ = initial value, *A*_2_ = final value, *X*_0_ = center value, dx = time constant. Control data from 10 other IHCs not subject to blockers (gray) are fit with a straight line. Blocked data significantly different from control. But there was no significant difference in time course or degree of block at 20 min between RgIA-5474 and Cy3-RgIA-5727. Inclusion of 0.1 mg/ml bovine serum albumin (BSA) prevented surface absorption of peptide and significantly accelerated block onset (green).

Intracellular recordings were made from cochlear hair cells to examine the effect of Cy3-RgIA-5727 or RgIA-5474 on native α9α10-containing nAChRs. Electrical stimulation evoked transmitter release from efferent axons that make synapses on IHCs in the first 2 weeks after birth in mice. With the IHC membrane voltage-clamped to −90 mV, evoked release of ACh from efferent contacts caused inward currents of 20–100 pA that decayed over 50–100 ms. The compound current through the nAChR and associated calcium-dependent potassium channels is entirely inward at −90 mV. The control and experimental values for synaptic currents were determined by averaging responses during prolonged trains of shocks at 1 or 5 Hz. Then RgIA peptide analogs were applied by bath perfusion. The average synaptic response declined over the course of 15–30 min of peptide perfusion (Figure 9A). Two-way ANOVA showed highly significant time-dependent block for all peptides compared to control (*p* < 0.0001, *F* = 6.83, df = 9). The time course of block by Cy3-conjugated RgIA-5727 appears to be slower but was not statistically different from that for RgIA-5474 (*p* = 0.582, *F* = 0.661, df = 3) and at 20 min there was no significant difference in the extent of block (0.32 ± 0.24) compared to that for RgIA-5474 (0.19 ± 0.13, two-tailed unpaired *t*-test, *p* = 0.29, *t* = 1.12, df = 8). There was no reversal of block in 12 cells with washing for as long as 50 min. Although only two experiments, co-application with BSA significantly accelerated the onset of block by RgIA-5474 (*p* < 0.019, *F* = 6.61, df = 1). BSA coats surfaces to reduce non-specific absorption of RgIA-5474.

Since the reduction of current in the presence of RgIA-5474 or Cy3-RgIA-5727 came on slowly and was essentially irreversible, an alternative interpretation is that the intracellular recording was simply failing. While that cannot be ruled out for any individual recording, “leak” current, an indication of recording quality, did not correlate with reduced postsynaptic current among all recordings in RgIA analogs. A collection of intracellular recordings of equivalent length shows that synaptic responses can be well-maintained in the absence of blockers (Figure 9B). Also, as found previously (Vattino et al., 2020; Zhang et al., 2020) ω-conotoxin GVIA blocked voltage-gated calcium channels in the efferent terminals over the course of 15–20 min, suggesting that conopeptides only slowly penetrate intact cochlear tissues.

## Discussion

Peptides found in animal venom have proven useful for the study of many ion channels and neurotransmitter receptors. Cone snails capture their aquatic prey using a sophisticated arsenal of conotoxins. These peptides are notable for subtype specificity. Here we describe the development of a fluorescently tagged analog of the peptide RgIA from the “royal cone,” *C. regius*. Substitutions in the native peptide sequence resulted in desirable ligand properties of high potency and slow off-rate kinetics (Romero et al., 2017; Gajewiak et al., 2021).

Reporter groups have traditionally been added to peptide ligands by means of crosslinking chemical groups to primary amines. Initial attempts at using *N*-hydroxysuccinimide ester dyes to conjugate to the N-terminus of the peptide resulted in low yields (data not shown) though on-resin strategies can substantially improve yields (Muttenthaler et al., 2020). We therefore added an alkyne group to the N-terminus of the peptide to enable reaction with Cy3-azide. We demonstrated that the Cy3-modified ligand potently and selectively blocks mouse nAChRs expressed in *Xenopus* oocytes. The click chemistry paradigm used for the synthesis described in this study should enable other reporter groups to be readily reacted with the alkyne-bearing RgIA peptide analog thus enabling a potential suite of nAChR-targeted ligands.

The binding of Cy3-RgIA-5727 to α9α10-containing nAChRs expressed in *Xenopus* oocytes is supported by site-specific binding of Cy3-RgIA-5727 onto cochlear hair cells in patterns corresponding to the known contacts of cholinergic efferent neurons. Furthermore, fluorophore conjugation does not prevent binding and blocking activity of RgIA analogs in oocytes or in hair cells. Thus, Cy3-RgIA-5727 can serve as a valuable tool for studying native α9α10-containing nAChRs in hair cells of the inner ear or other tissues. Although best characterized in cochlear hair cells, α9 nAChR subunits have been reported in a variety of specialized tissues including immune cells, pituitary gland, lung epithelium, certain cancers, sperm, and bladder (Peng et al., 2004). The function of the formed nAChRs in these tissues remains poorly understood. Block of α9a10 nAChRs is associated with analgesia in nerve trauma and chemotherapy-induced neuropathic pain models. KCP-506, an α9α10 nAChR antagonist, has recently entered human clinical trials for the treatment of neuropathic pain. α9 subunit-null mice are resistant to the therapeutic effects of α9α10 nAChR antagonists (Romero et al., 2017). The mechanism by which α9α10 antagonists produce analgesia is an area of active investigation. α9-containing nAChRs have recently been reported in subsets of immune cells where they have been shown to modulate the release of the inflammatory cytokine interleukin-1β. The precise composition of these immune cell nAChRs is unknown but may include the presence of α10 as well as α7 subunits (Hecker et al., 2015; Richter et al., 2016, 2018, 2020; Siebers et al., 2018; Zakrzewicz et al., 2019). The availability of Cy3-RgIA-5727 should serve as a novel probe for the study of these and related receptors.

At the same time, the present study highlights some cautionary rules for the use of this compound. While Cy3-RgIA-5727 blocked heterologous α9α10-containing nAChRs in *Xenopus* oocytes with picomolar affinity, it was necessary to use nanomolar concentrations to produce block in cochlear tissue over the limited timeframe of such *ex vivo* experiments. This difference may reflect diffusional constraints of the intact tissue, compared to fully exposed cells such as the oocyte.

Specific labeling at cholinergic synapses was clearly demonstrated by utilizing α9-subunit-null mice. However, residual non-specific binding in tissue from knockout mice and in tissue pre-bound by unlabeled RgIA analog suggests that Cy3-RgIA-5727 is sticky and may be absorbed in intact tissue. This is consistent with the hydrophobic nature of the Cy3 fluorophore. Coupling of Cy3 to the RgIA analog resulted in a peptide that had a rightward shift in reversed-phase HPLC elution time consistent with increased hydrophobicity. Cy3-RgIA-5727 retained the desirable property of slow reversibility. This allowed the target tissue to be washed extensively after exposure to ligand. The slow off-rate kinetics may prove valuable for other applications such as flow-cytometry that require extended ligand binding time.

## Data Availability Statement

The raw data supporting the conclusions of this article will be made available by the authors, without undue reservation.

## Ethics Statement

The animal study was reviewed and approved by University of Utah Animal Care and Use Committee (Protocol #20-07003) and Johns Hopkins University Animal Care and Use Committee (Protocol #MO19M478).

## Author Contributions

FF, YZ, PV, JG, and TG designed, conducted, and analyzed the experiments and edited the manuscript. EG directed the project and edited the manuscript. PF and JM directed the project, analyzed the results, and wrote and edited the manuscript. All authors contributed to the article and approved the submitted version.

## Conflict of Interest

Certain conopeptides have been patented by the University of Utah; JG and JM are inventors on these patents. The remaining authors declare that the research was conducted in the absence of any commercial or financial relationships that could be construed as a potential conflict of interest.

## Publisher’s Note

All claims expressed in this article are solely those of the authors and do not necessarily represent those of their affiliated organizations, or those of the publisher, the editors and the reviewers. Any product that may be evaluated in this article, or claim that may be made by its manufacturer, is not guaranteed or endorsed by the publisher.
